# Incidence of brain metastases in patients with early HER2-positive breast cancer receiving neoadjuvant chemotherapy with trastuzumab and pertuzumab

**DOI:** 10.1038/s41523-022-00380-7

**Published:** 2022-03-22

**Authors:** Emanuela Ferraro, Jasmeet Singh, Sujata Patil, Pedram Razavi, Shanu Modi, Sarat Chandarlapaty, Andrea V. Barrio, Rachna Malani, Ingo K. Mellinghoff, Adrienne Boire, Hannah Y. Wen, Edi Brogi, Andrew D. Seidman, Larry Norton, Mark E. Robson, Chau T. Dang

**Affiliations:** 1 Breast Cancer Service, Department of Medicine, Memorial Sloan Kettering Cancer Center, New York, NY USA; 2 Breast Cancer Service, Department of Medicine, Memorial Sloan Kettering Cancer Center, Weill Cornell Medicine College, New York, NY USA; 3 Breast Cancer Service, Department of Surgery, Memorial Sloan Kettering Cancer Center, New York, NY USA; 4 Brain Tumor Center, Department of Neurology, Memorial Sloan Kettering Cancer Center, New York, NY USA; 5 Brain Tumor Center, Human Oncology and Pathogenesis Program, Department of Neurology, Memorial Sloan Kettering Cancer Center, New York, NY USA; 6 Department of Pathology, Memorial Sloan Kettering Cancer Center, New York, NY USA; 7 Present Address: Department of Quantitative Health Sciences, Cleveland Clinic, Cleveland, OH USA

**Keywords:** Cancer epidemiology, Breast cancer, Breast cancer

## Abstract

The addition of pertuzumab (P) to trastuzumab (H) and neoadjuvant chemotherapy (NAC) has decreased the risk of distant recurrence in early stage HER2-positive breast cancer. The incidence of brain metastases (BM) in patients who achieved pathological complete response (pCR) versus those who do not is unknown. In this study, we sought the incidence of BM in patients receiving HP-containing NAC as well as survival outcome. We reviewed the medical records of 526 early stage HER2-positive patients treated with an HP-based regimen at Memorial Sloan Kettering Cancer Center (MSKCC), between September 1, 2013 to November 1, 2019. The primary endpoint was to estimate the cumulative incidence of BM in pCR versus non-pCR patients; secondary endpoints included disease free-survival (DFS) and overall survival (OS). After a median follow-up of 3.2 years, 7 out of 286 patients with pCR had a BM while 5 out of 240 non-pCR patients had a BM. The 3-year DFS was significantly higher in the pCR group compared to non-pCR group (95% vs 91 %, *p* = 0.03) and the same trend was observed for overall survival. In our cohort, despite the better survival outcomes of patients who achieved pCR, we did not observe appreciable differences in the incidence of BM by pCR/non-pCR status. This finding suggests that the BM incidence could not be associated with pCR. Future trials with new small molecules able to cross the blood brain barrier should use more specific biomarkers rather than pCR for patients’ selection.

## Introduction

Central nervous system (CNS) is a common site of distant recurrence that affects prognosis and quality of life of HER2-positive breast cancer (BC) patients^1^. The reported cumulative incidence of brain metastases (BM) in HER2 positive BC is higher than in other subtypes suggesting that HER2 positive cancer cells have a specific tropism for the CNS^2,3^. The advent of different anti-HER2 agents and the implementation of local approaches such as stereotactic radiosurgery (SRS) has significantly improved the prognosis of HER2- positive BC patients with BM. However, BM still presents multiple challenges for optimal management, especially in the scenario of progression despite loco-regional therapies. New oral HER2 tyrosine kinase inhibitors (TKIs) including neratinib and tucatinib, have demonstrated CNS activity, and have been recently approved by Food and Drug Administration (FDA) in metastatic setting^4,5^. In early stage, neratinib is currently approved as single agent after trastuzumab-based adjuvant therapy^6^ and tucatinib is still under investigation in high-risk patients in combination with T-DM1 (NCT04457596).

In stages I-III BC, the CNS recurrence rate is reported around 2–4% of patients receiving trastuzumab and/or pertuzumab- based adjuvant treatments as first site of recurrence in a follow-up range of 3–5 years^7–9^. Few studies have reported the rate of BM in early-stage breast cancer patients treated with neoadjuvant chemotherapy (NAC). The addition of pertuzumab to trastuzumab and chemotherapy in HER2-positive BC has resulted in an improvement of pathologic complete response (pCR) rate after NAC^10^. Currently, the rate of BM and the predictive role of pCR on the risk of CNS seeding is unknown in patients receiving double blockade with trastuzumab and pertuzumab (HP) in preoperative setting. The interest in understanding the incidence of CNS recurrence in a real-life population arises from the necessity to shape new strategies to reduce the risk of BM in patients with early-stage BC. The aim of this study was to assess the incidence of BM in patients receiving HP-containing NAC and to compare rates of BM stratified by pCR status.

## Results

### Study population

**Fig. 1:**
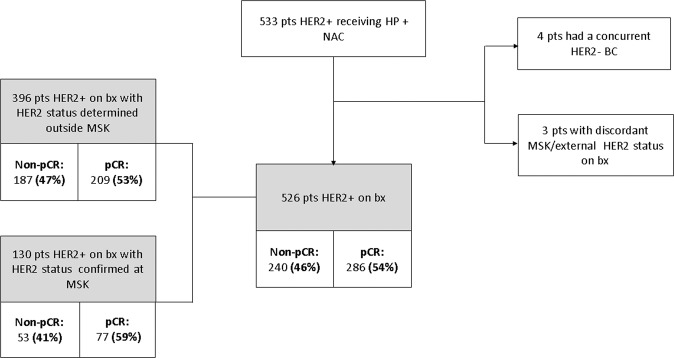
Diagram of patients’ selection. This schema represents a consort diagram of the study and provides the patients selection based on HER2 status on biopsy and response to neoadjuvant treatment (pCR versus non-pCR). Notes: HER2+: HER2-positive, HER2−: HER2-negative, pts: patients; BC: breast cancer; bx: biopsy.

Overall, 533 patients with stage I-III HER2 positive breast cancer treated with NAC followed by surgery at MSKCC were identified. Cases with a concomitant HER2 negative BC (*n* = 4) and discordant HER2 status (internal versus external) (*n* = 3) were excluded. Among the study population (*n* = 526), 130 patients had preoperative HER2 status confirmed at MSKCC (Fig. 1). All clinicopathological features are described in Table 1. A pCR was achieved in 286/526 (54.4%) of cases, whereas 240/526 (45.6%) had residual disease. The majority of the patients (278/286, 97% in pCR and 226/240, 94% in non-pCR group) had a poorly differentiated breast carcinoma. The clinical stage II was the prevalent stage at the time of diagnosis (74% and 72% in pCR and non-pCR groups, respectively). In both groups, most of patients received dose dense chemotherapy with doxorubicin/cyclophosphamide followed by paclitaxel plus HP (AC-THP) as NAC (90% vs 86% in pCR and non-pCR respectively). Anthracycline-free therapy with docetaxel, carboplatin plus HP (DCbHP regimen) was administered in less than 10% of cases, in both groups. A minority of patients received vinorelbine and gemcitabine, when taxane was contraindicated (Table 1).

**Table 1:** Patients’ characteristics.

	Overall population (*n* = 526)			MSKCC HER2-status confirmed on biopsy (*n* = 130)		
	pCR *n* (%) *n* = 286 (54.4)	non-pCR *n* (%) *n* = 240 (45.6)	*p*-value*	pCR *n* (%) *n* = 77 (59.2)	non-pCR *n* (%) *n* = 53 (40.7)	*p*-value*
Median age, years (range 26–82)	50 (24-84)	50 (26-87)	0.078	50 (28-82)	49 (26-76)	
Menopausal status			0.2			
Pre	145 (50.7)	137 (57)		38 (49)	28 (53)	
Post	141 (49.3)	103 (43)		39 (51)	25 (47)	
Clinical stage			0.010			
I	16 (5.6)	3 (1)		1 (1.3)	36 (68)	
II	212 (74.2)	173 (72)		58 (75.3)	15 (28)	
III	58 (20.2)	64 (27)		18 (23.4)	2 (4)	
Clinical T			**0.042**			
Tx	6 (2)	0		4 (5.2)	0	
T1	47 (16.4)	23 (9.6)		6 (7.8)	3 (5.7)	
T2	171 (59.7)	153 (63.7)		47 (61)	38 (71.8)	
T3	41(14.3)	44 (18.3)		16 (20.8)	9 (16.9)	
T4	15 (5.2)	15 (6.3)		3 (3.9)	2 (3.8)	
T4d	6 (2)	5 (2.1)		1 (1.3)	1 (1.8)	
Lymph nodes involvement (clinical staging)	114 (39.8)	84 (35)	0.3	27 (35)	19 (35.9)	
N0	147 (51.5)	128 (53.4)		42 (54.5)	27 (50.9)	
N1	15 (5.2)	21 (8.7)		6 (7.8)	5 (9.5)	
N2	10 (3.5)	7 (2.9)		2 (2.7)	2 (3.7)	
N3						
HER2 status on biopsy			**<0.001**			
IHC 3+	268 (94)	164 (68.3)		76 (99)	27 (51)	
FISH amplified	18 (6)	76 (31.7)		1 (1)	26 (49)	
HR status on biopsy			**<0.001**			
Positive	153(54)	192 (80)		41 (52)	38 (70)	
Negative	133 (46)	48 (20)		36 (48)	15 (30)	
Histology on biopsy			NA**			
Ductal	284 (99.3)	236 (98.3)		76 (99)	52 (99)	
Lobular	2 (0.7)	4 (1.7)		1 (1)	1 (1)	
Differentiation			0.2			
Well/moderated	8 (2.7)	14 (5.8)		6 (8)	13 (24.5)	
Poorly differentiated	278 (97.3)	226 (94.2)		71 (92)	40 (75.5)	
NAC regimens			0.4			
ACTHP	256 (89.5)	206 (85.8)		69 (90)	48 (90)	
DCbHP	18 (6.3)	22 (9.2)		3 (4)	2 (4)	
Other	12 (4.2)	12 (5)		5 (6)	3 (6)	
Type of breast surgery			0.70			
Mastectomy	153(53.5)	134 (55.8)		41 (53)	32 (61)	
Lumpectomy	131 (45.8)	106 (44.2)		35(45)	21 (39)	
Axillary dissection***	2 (0.7)	0		1 (2)	0	
Type of axillary surgery			**<0.001**			**<0.001**
Dissection	20 (7)	85 (35)		9 (12)	22 (42)	
SNLB	266 (93)	155 (65)		68 (88)	31 (58)	
Radiation treatment			**0.009**			
Yes	244 (85.3)	223 (92.9)		39 (50.6)	36 (67.9)	
No	42 (14.7)	17 (7.1)		38 (49.4)	17 (32.1)	
Adjuvant anti-HER2 therapy						
HP	283 (99)	227 (94.6)	NA	77 (100)	51 (96)	
HP → neratinib	0	3 (1.3)		0	0	
TDM1	0	8 (3.3)		0	1 (2)	
H	3 (1)	2 (0.8)			1 (2)	
Adjuvant endocrine treatment	HR + = 153	HR + = 192	NA	HR + = 41	HR + = 38	
AI	62 (40.5)	93 (48.4)		20 (48.7)	24 (63.2)	
TAM	61 (39.8)	54 (28.2)		15 (36.6)	10 (26.3)	
AI + LHRH	14 (9.2)	31 (16.2)		1 (2.4)	0	
TAM + LHRH	1 (0.7)	7 (3.6)		0	0	
No ET****	15 (9.8)	7(3.6)		5 (12.3)	4 (10.5)	

Bold values indicates statistically significant *p* values < 0.05.

*L* line, *NAC* neoadjuvant chemotherapy, *ACTHP* doxorubicin, cyclophosphamide, paclitaxel; trastuzumab; pertuzumab; *HT* hormonotherapy, *DCbHP* docetaxel, carboplatin, trastuzumab, pertuzumab, *HR* hormone receptor, *AI* aromatase inhibitors, *TAM* tamoxifen, *T* primary tumor, *NA* not applicable.

*Statistical tests performed: chi-square test of independence; *t*-test.

**NA due to small sample sizes in a category.

***In cases of Tx, the patients received just axillary dissection. These cases are not included in the statistical analysis because all the cases are related to the pCR group.

****No ET: patients with HR-positive tumors who did not receive endocrine treatments due to decline or clinical decision in case of low-ER and PR expression

Patients who achieved pCR compared to those did not, had more frequently HR negative tumors (46% vs 20% *P* < 0.001) and more often had HER2 overexpression by IHC (3 + ) (94% vs 68%, *P* < 0.001). In the pCR group, the proportion of patients who received axillary dissection was lower than in the non-pCR group (7% vs 35% *P* < 0.001). These results were consistent between overall patients and the subset of patients with HER2 status on pre-NAC biopsy verified at MSKCC (Table 1).

### Disease-free survival events

After a median follow-up of 3.2 years (range 0.4–5.5), 36 DFS events occurred in the study population, 14 in the pCR group and 22 in the non-pCR group (Table 2). Among pCR patients with recurrences, 4/14 had locoregional recurrence, 9/14 had distant recurrence of which 7 had only BM, 1 visceral metastasis (lung) and 1 non-visceral metastasis (thoracic lymph nodes). One patient in the pCR group died of unknown cause. The loco-regional breast disease events included 2 contralateral breast cancer, 1 ipsilateral lymph nodal, 1 invasive breast cancer and 1 DCIS relapses. Conversely, almost the totality (17/22) of DFS events of the non-pCR group were distant relapse with 7/22 patients with visceral recurrence in the liver and adrenal glands, 5/22 skin and lymph nodes recurrence and 5/22 with a brain only recurrence.

**Table 2:** Disease-free survival events: pCR versus non-pCR patients.

	Overall population (*n* = 526)		MSKCC HER2-status (*n* = 130)	
	pCR *n* = 286	non-pCR *n* = 240	pCR Tot: 77	non-pCR Tot: 53
**DFS events**	14	22	8	5
**Locoregional recurrence**	4	3	4	0
Breast	1	2	1	—
Regional lymph nodes	2	—	2	—
DCIS	1	1	1	—
**Distant recurrence**	9	17	4	5
Brain only	7	5	3	2
Visceral disease	1	7	1	2
Non-visceral disease	1	5	0	1
Death*	1	2	0	0

*death without prior recurrence events.

*pCR* pathological complete response, *DCIS* ductal carcinoma in situ.

### Brain metastases incidence

**Fig. 2:**
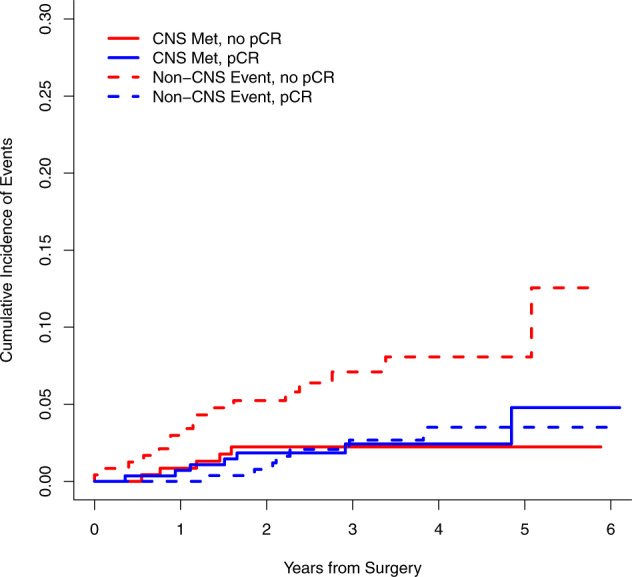
Cumulative incidence of CNS recurrence events stratified by pCR versus non-pCR. The continuous lines represent the estimated incidence of CNS events and the dashed lines the incidence of non-CNS events in non-pCR (red lines) and pCR group (blue lines), respectively.

There was a total of 7/286 (2.4%) BM events without other extracranial sites of disease in the pCR group, and 5/240 (2%) in the non-pCR group after a median follow-up of 3.2 years. We did not observe any meaningful visual differences in the cumulative incidence curves for BM for the two groups (Fig. 2). Among overall BM events (*n* = 14), 12 occurred as a first event of recurrence. In the pCR group the totality of patients developed BM as first events of recurrence, while in the non-pCR group 2 patients had BM as second event. The median time to development BM observed in our population was 19 (range 4–58) months and 6.5 (range 6.5–17) months in the pCR and non-pCR group, respectively.

Most of the patients had one or two brain lesions in both pCR and non-pCR groups, who underwent surgical resection followed by stereotactic radiation of the tumoral bed or radiosurgery alone. Whole brain radiation was delivered to 3 patients in the pCR group and in 1 patient in the non-pCR group. One patient of the pCR group and one of the non-pCR group had an extensive disease with severe symptoms that did not improve after local treatment. The majority of the patients received a first line systemic treatment for metastatic disease, except for two patients who continued adjuvant HP after the local treatment (Table 3). The baseline characteristic of patients who developed brain BM were homogeneous regardless the pCR status. Eleven out of 14 patients had clinical stage III disease while 3/14 stage II. In the pCR group, 5/7 patients had HR + /HER2 + disease while 3/7 patients in the non-pCR group. Only 2 patients in the pCR group and 5 patients in the non-pCR group had HR-HER2 + disease at the time of diagnosis.

**Table 3:** Description of patients with brain metastases.

	Age^a^ (yrs)	Clinical stage	HR status	Time to BM	Symptoms^b^	Brain as first site of recurrence (Y/N)	Need of hospitalization (Y/N)^c^	N of lesions as first BM event	Local Treatment	Systemic treatment	Time to 1° CNS POD or death
**pCR**	61	cT2 N0	+	44 mo	seizures and visual changes	Y	Y	1	Surgery and SRT	Cape/Lap	19 mo
	40	cT2 N1	+	35 mo	cephalgia and vomiting	Y	Y	1	Surgery and SRT	Cape/Lap	12 mo
	36	cT3N2	+	14 mo	ataxia, aphasia and fatigue	Y	Y	> 10	WBRT	BSC	6 mo
	49	cT1N3	−	4 mo	cephalgia	Y	Y	3	WBRT	HP	2 mo
	48	cT4N1	+	11 mo	vertigo and nausea	Y	Y	> 10	WBRT	Cape/Lap	10 mo
	66	cT4N1	+	18 mo	ataxia	Y	N	> 10	proton CSI	Cape/Lap	11mo
	55	cT4N1	+	19 mo	aphasia	Y	Y	2	SRT	HP	NR
**Non-pCR**	44	cT2 N3	+	15 mo	ataxia	Y	N	1	Surgery and SRT	LET + H	4 mo
	47	cT4d N1	−	9 mo	facial drop	Y	N	2	SRS	HP	16 mo
	61	cT2N1	+	19 mo	ataxia	Y	Y	1	Surgery and SRT	ANA + H	7 mo
	48	cT4N1	−	17 mo	seizures	Y	Y	2	Surgery and SRT	Cape/Lap	NR
	52	cT3N1	−	6 mo	vertigo	Y	Y	1	Surgery and SRT	BSC	2 mo
	47	cT3N0	+	55 mo	nausea	N	Y	2	SRS	TH	10 mo
	44	cT4N2	−	14 mo	cephalgia	N^d^	Y	> 10	WBRT	THP	5 mo

*pCR* pathological complete response, *yrs* years, *mo* months, *BM* brain metastases, *HR* hormone receptors, (+):ER and/or PR > 1%, (−): ER and/or PR < 1%, *CNS* central nervous system, *POD* progression of disease, *H* trastuzumab, *HP* trastuzumab and pertuzumab, *Cape* capecitabine, *T* paclitaxel, *Lap* lapatinib, *ANA* anastrozole, *LET* letrozole, *SRT* stereotactic radiotherapy, *SRS* stereotactic radiosurgery, *NA* not applicable, *NR* not reached, *BSC* best supportive care, *WBRT* whole brain radiation, *proton CSI* proton cranio-spinal irradiation.

^a^Age at the time of brain progression.

^b^Brain metastases symptoms suggesting need of brain radiological assessment.

^c^Need of hospitalization for the management of neurological symptoms at the time BM relapse.

^d^BM have been discovered one month after the extracranial disease (liver/chest wall).

### Survival outcomes

**Fig. 3:**
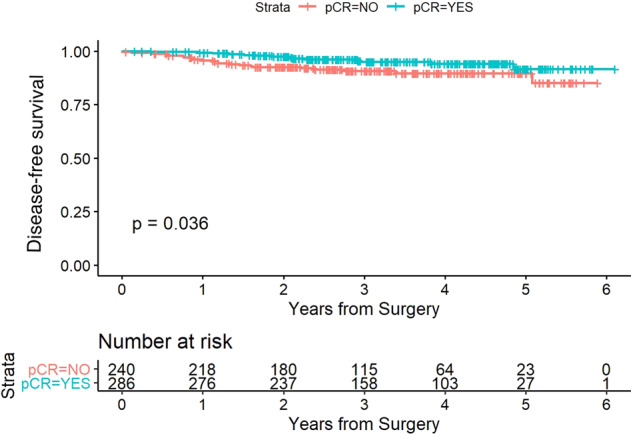
Disease-free survival stratified in pCR versus non-pCR groups. The red and blue curves show the estimated disease-free survival of the patients in the non-pCR group and pCR group, respectively.

**Fig. 4:**
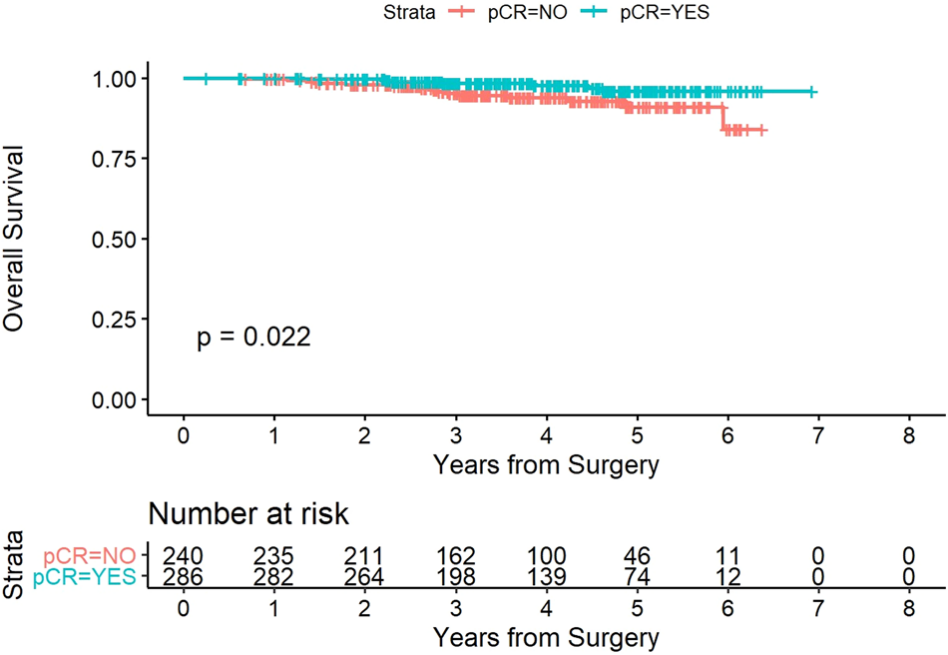
Overall survival stratified in pCR versus non-pCR groups. The red and blue curves show the estimated overall survival of the patients in the non-pCR group and pCR group, respectively.

Regarding survival outcomes, the 3-year DFS was 91% (95% CI 87–95%) in the non-pCR group and 95% (95% CI 92–98%) in the pCR group (*p* = 0.03). The 3-years OS was 95% (95% CI 92–98) in the non-pCR group and 98% (95% CI 97–100) in the pCR group (log-rank *p* = 0.02) (Figs. 3 and 4).

## Discussion

In our cohort, the absolute rate of BM was similar between pCR and non-pCR patients, 2.4% and 2%, respectively, with the median follow-up of 3.2 years. The median time of BM relapse was 19 versus 6.5 months in patients with pCR versus non-pCR, respectively. Notably, in the pCR group, 30% of DFS events were local recurrence and almost the totality of patients with distance recurrence had BM as first recurrence event. Conversely, in the non-pCR group, the majority recurrence events were distant relapse with extra-cranial sites of disease. A reasonable explanation of the predominance of brain relapse in patients who achieve pCR, could be that anti-HER2 antibodies currently used in early stage are extremely active to clear the extracranial compartments from micro-metastatic disease, inducing a selection of resistant clones with brain tropism. Patients with residual disease may have a more resistant and heterogeneous disease with more variable clonal selection.

Additionally, the combination of chemotherapy and HP was associated with pCR rate of 54% and excellent survival outcome compared with patients who did not achieve pCR, consistently with literature data^10–13^. Patients with HR-negative tumors and HER2 IHC 3+ were more likely to achieve pCR, as previously reported by investigators at our institution^14^. These data were confirmed in the subgroup of patients with HER2 status performed at MSKCC, suggesting reliability of the HER2 status of our entire population. As expected, a statistically significant difference in terms of 3-year DFS and OS was observed in favor of the pCR group.

Currently, HP dual blockade plus chemotherapy has become the standard of care in patients with early stage HER2- positive BC^15^. Several trials have demonstrated that the combination of HP with standard chemotherapy, can lead to a pCR rate of about 60%^11,16–18^. At individual level, patients with pCR have better outcomes than those with non-pCR, most notably in those with HER2-positive hormone receptor-negative and triple negative diseases^19,20^. In our study, the similar BM rates between pCR and non-pCR groups suggest that effective therapies that cross the blood brain barrier are needed. Our data is consistent with the results of the APHINITY trial^9^ that showed similar BM rate in the HP and standard arm of 1.9% and 1.8%, respectively.

In addition, there appears to be no association between NAC response and BM event rates although the absolute number of events in our population is too small to draw conclusion. Notably, a pooled analysis of GeparQuinto and GeparSixto^21^, which included both early HER2-positive BC treated with trastuzumab or lapatinib based-regimes and triple negative tumors, showed similar conclusions. BM as first site of metastatic disease occurred more frequently than other distant metastases in patients with the pCR (15% vs 9%), suggesting no correlation between response to NAC and BM recurrence.

The rate of BM reported in our study was consistent with the rates registered in controlled prospective trials in early stage. For instance, in the KATHERINE trial^22^, testing trastuzumab-DM1(T-DM1) in the post-neoadjuvant setting for patients with non-pCR, the incidence of BM after 3-years of follow-up was 5.9% and 4.3% in patients who received T-DM1 and trastuzumab, respectively. T-DM1 as well as other anti-HER2 agents approved in the treatment of early-stage BC including trastuzumab^23–27^ and neratinib^6^ appear to have no impact on the risk of CNS recurrence. In the ExteNET trial^6^, patients were treated with adjuvant trastuzumab alone or sequential neratinib for one year. Regardless of the assigned therapy in the trial, the rate of CNS recurrence was 1% in each arm, after a median follow-up of 5.2 years. Nevertheless, a recent unplanned subgroup analysis showed a decreased cumulative incidence of BM in neratinib arm compared to placebo arm in a subset of hormone receptor positive, HER2 positive BC patients who started neratinib ≤1 year from the end of adjuvant trastuzumab^28^.With regards to lapatinib, ALTTO^29^ and NEOALTTO studies^30^ showed that lapatinib alone or in combination with trastuzumab in adjuvant and neoadjuvant setting did not reduce the rate of brain recurrence.

Due to the low CNS penetrance, bioavailability or activity of the approved anti-HER2 compounds, the brain is a sanctuary for metastatic disease. More recently, tucatinib, a new TKI has demonstrated activity on BMs in patients with advanced pretreated HER2-positive BC and it has been FDA approved. In the phase I study^31^, the combination of tucatinib and ado-trastuzumab emtansine led to 36% of response in BM lesions. The study of Tucatinib vs. Placebo in Combination With Capecitabine and Trastuzumab in Patients With Advanced HER2 + Breast Cancer (HER2CLIMB trial)^5^, showed a benefit in terms of progression-free survival (PFS) and OS in patients who received tucatinib in combination with trastuzumab and capecitabine. In patients with BMs, the estimated PFS at 1 year was 24.9% (95% CI, 16.5 to 34.3) in the tucatinib-combination arm and 0% in the placebo arm. Additionally, the reported CNS-PFS was 9.9 months in the tucatinib arm versus 4.2 months in the control arm with a reduction of the risk of death by 42% in the tucatinib arm (HR: 0.58)^32^. These data are particularly relevant, because BM still represent a source of morbidity and mortality in patients with HER2-positive BC. Indeed, with incremental improvement with modern systemic treatment in reducing breast cancer recurrences, the management of BM has become an essential component of disease control and quality of life of patients. In the treatment of early stage disease, new escalating approaches have not been associated with a decrease in BMs^6,9,22^. Clinical trials in early-stage setting should explore novel drugs and strategies that may impact on BM recurrence, including with an exploratory focus on detection of BMs in asymptomatic patients. In our cohort, patients who achieved pCR, despite the better overall outcome, seem to be still at risk of brain recurrence and for this reason, they could benefit from escalating post-neoadjuvant treatment with new TKIs as well as non-pCR patients. Moving forward, trials exploring early detection of BMs in patients with early BC could help to optimize the management of brain recurrence. To date no radiological screening is recommended by ASCO^33^ and NCCN guidelines^34^ and potential benefit of early detection of asymptomatic BM is being explored in advanced setting (NCT03881605, NCT04030507, NCT03617341).

To our knowledge, this is the first sizeable analysis of patients treated with HP-based therapy, off-study, in neoadjuvant setting with a focus on BM recurrence. However, the study has several limitations including the retrospective nature, the single center setting, along with a modest number of BM events. We await a longer follow-up to see if the rate of BM increases over time in the two groups. Additionally, only a few patients with residual disease received T-DM1 as adjuvant treatment, as most of the patients were on adjuvant treatment before the approval of T-DM1 by the Food and Drug Administration in this setting. The strength of our study is that it includes a large cohort of patients who received HP based- NAC, with more than 90% receiving dose-dense AC-THP. Despite the inclusion of a large proportion of patients with external HER2 status, the analyses on the subgroup with MSKCC-verified HER2 positivity were similar for the entire population. Furthermore, this study provides a valid evidence about the incidence of brain recurrence after HP given preoperatively and continued after surgery. Indeed, neoadjuvant prospective trials did not report the BM event rates separately from extra-cranial distant recurrence events^35–37^.

We reported for the first time the incidence of brain recurrence in patients with pCR versus non-pCR after HP combined with chemotherapy, that seems to be not associated with response to NAC. However, a longer follow-up is awaited to confirm these results. Our findings support the investigation of new molecules with high CNS bioavailability in early stage HER2-positive BC in order to evaluate a possible role of these agents to prevent brain recurrence. Research on specific biomarkers of CNS seeding is crucial to better select the population that might benefit from an escalating post-neoadjuvant treatment. Current data do not support pCR being one of those biomarkers.

## Methods

### Patients’ selection

We reviewed the medical records of consecutive early stage HER2-positive breast cancer patients from the hospital cancer registry at Memorial Sloan Kettering Cancer Center (MSKCC), between September 1, 2013 to November 1, 2019. Follow-up data were obtained until June 30, 2020.

We included patients with HER2-positive breast cancer who received HP in the neoadjuvant setting. Trastuzumab and pertuzumab were administered in combination with standard chemotherapy and for at least one cycle before surgery. Surgery was performed within 6 weeks after NAC. Adjuvant treatments, including endocrine therapy and anti-HER2 therapy, were offered according to physician’s choice. Radiotherapy (RT) was offered as per standard of care. HER2 positivity was defined according to ASCO-CAP guidelines^38,39^, either as HER2 overexpression (3 + ) by immunohistochemistry (IHC) or gene amplification by fluorescence in situ hybridization (FISH). Hormone receptor (HR) status—estrogen receptor (ER) and progesterone receptor (PR)—was assessed by IHC and considered positive when ≥1% of cancer cell nuclei were stained^40^. All cases were reviewed at MSKCC by dedicated breast pathologists and the diagnosis was verified for all cases. HER2 and HR status were performed on biopsy samples at MSKCC in a limited subset of patients. For the cases tested at MSK, pre-diluted VENTANA anti-HER2/neu (4B5) Rabbit Monoclonal Primary Antibody has been used to determine HER2 status. For those patients whose receptor status were based on a biopsy performed at another institution, those with HER2 assessment repeated and verified at MSKCC were included in the sensitivity analysis. During post-treatment follow-up, brain imaging with MRI was performed if there were any concerning signs or symptoms. Patients with leptomeningeal disease only were excluded. If patients experienced BM, they were treated with standard of care per physician’s discretion, including surgery and/or radiotherapy as clinically indicated.

### Ethics

The study has been conducted in accordance with the Good Clinical Practice guidelines and the Declaration of Helsinki. Data has been collected and analyze after receiving approval from the MSKCC Institutional Review Board under the number 20-436. All patients reviewed in this study were consented to an institutional protocol, which gives investigators access to their clinical data for research purposes.

### Objectives

The primary endpoint is the incidence of BM when it was first site of relapse in pCR and non-CR group. The pCR was defined as absence of residual invasive carcinoma in breast and axilla (ypT0/is ypN0). Disease free survival (DFS) and overall survival (OS) are secondary endpoints. DFS is defined as the interval between the date of surgery and date of any breast disease event, date of death from any cause, or, in case of non evidence of disease (NED), date of last follow-up. Breast disease events included loco-regional recurrence (ipsilateral breast recurrence of invasive carcinoma, regional-nodal recurrence, contralateral invasive breast cancer and DCIS) and distant recurrence (both CNS and non-CNS). OS, defined as the interval between diagnosis date and date of death from any cause or if alive, date of last follow-up, was also evaluated.

### Statistical analysis

The incidence of BM was estimated using the cumulative incidence function^41^ and compared by pCR status using the Gray test^42^. DFS and OS were analyzed using the Kaplan-Meier method and differences assessed using the log-rank test. Differences between clinicopathological features and pCR status was evaluated using chi-square test and *t*-test. Any *p*-value less than 0.05 was deemed to be statistically significant. In a sensitivity analysis, all analyses were done on the whole population (*N* = 526) and on the subgroup of patients who had a verified HER2 status at MSKCC (*N* = 130).

### Reporting summary

Further information on research design is available in the Nature Research Reporting Summary linked to this article.

## Supplementary information

Reporting Summary

## Data Availability

All data analyzed in this study are included in this manuscript. The data supporting Table 1 as well as MRI imaging data are not publicly available in order to protect patient privacy but can be made available for non-commercial use only and on reasonable request from the corresponding author. For the data sharing, an agreement with the corresponding author about data usage is required.
